# Twitter data emotion analysis using Hadoop and metaheuristic optimized Graphical Neural Network

**DOI:** 10.3389/frai.2025.1672252

**Published:** 2025-10-23

**Authors:** Xiaohui Wang, Yang Li, Fangyuan Chen

**Affiliations:** ^1^School of Big Data, Qingdao Huanghai University, Qingdao, Shandong, China; ^2^College of Computer Science and Engineering, Shandong University of Science and Technology, Qingdao, China

**Keywords:** Hadoop, R-visualization, Twitter, movie reviews, optimization, Graphical Neural Network, rolling window, map reduce

## Abstract

This study applies the Hive framework within the Hadoop ecosystem for sentiment classification, focusing on emotion analysis of X data. After outlining Hadoop’s core advantages in large-scale unstructured data processing, the study focuses on using a Graphical Neural Network (GNN) for sentiment categorization of Twitter comments. To address the suboptimal performance of traditional GNNs due to trial-and-error hyperparameter tuning, the study introduces the Modified Elephant Herd Optimization (MEHO) algorithm—improved version of the standard EHO, to optimize the network’s weight parameters, hyperparameters, and feature subsets, ensuring a balance between exploration and exploitation. An automated dataset construction system has also been developed to reduce manual labeling effort and ensure consistency. Preprocessing techniques, including information entropy–based phrase ranking, further enhance data quality. To capture both semantic and statistical features of tweets, feature extraction methods such as Term Frequency–Inverse Document Frequency (TF–IDF) and Bag of Words (BoW) are integrated. Experimental results demonstrate that MEHO reduces premature convergence by 40% and improves classification accuracy by 6.1% compared with the standard EHO algorithm. The automated labeling system decreases manual effort by 80%, while entropy-based preprocessing increases phrase difficulty classification accuracy by 7%. This study provides an effective solution for social media emotion analysis; future research will explore multi-modal data fusion and optimize MEHO’s convergence speed for ultra-large feature sets.

## 1 Introduction

The rapid growth of social networking platforms like Twitter has significantly altered how individuals express their emotions and opinions, leading to large-scale unstructured data (Neethu and Rajasree, 2013). The internet has transformed how people communicate, using platforms such as blogs and social media, which has resulted in an overwhelming volume of data that is difficult to process manually (Mejova, 2009). However, the sheer volume of such information makes manual processing impossible. In this context, automated sentiment analysis plays a crucial role. Sentiment analysis, which is the computational process of determining the polarity (e.g., positive or negative) of a text to recognize a user’s opinion on a specific issue (Boiy et al., 2007), enables the extraction of actionable insights from large-scale textual data. Specifically, this study applies sentiment analysis to tweets about movie reviews, focusing on feature-based sentiment analysis. The objective is to identify and categorize nuanced emotions such as anger, longing, and love within these tweets. In the present study, a smaller sample of 3,500 tweets is used to validate the approach; the Hadoop-based architecture is adopted for its scalability and will be fully exercised in subsequent large-scale evaluations.

Analyzing X data is challenging due to its high volume, topic diversity, and the complexity of inferring underlying sentiments (Ennaji et al., 2015). When an organization aims to understand customer perspectives on a product, it becomes challenging to effectively monitor and record every individual viewpoint. In such cases, sentiment analysis becomes crucial. It refers to the process of determining the polarity of a given text, specifically recognizing the user’s opinion on a particular issue (Sheela, 2016). The use of sentiment analysis is very beneficial in gaining insight into an individual’s attitudes and reactions towards a certain subject matter. Most internet data is unstructured, making it challenging to process and extract useful information for analysis (Verma et al., 2015). Hadoop, with its MapReduce framework and Hadoop Distributed File System (HDFS), provides an effective solution for handling large datasets, including X data in JSON format (Yadav et al., 2016; Pujol et al., 2011). Hadoop’s ecosystem, including tools like Apache Flume for data extraction and Apache Hive for querying, supports the efficient handling of X data in JSON format (Chakradhar and Raghunathan, 2010). The analysis of the data stored in the HDFS is performed using the data access components provided by the Hadoop ecosystem, namely Apache Pig and Apache Hive. The Hadoop Ecosystem has two primary data access components, namely Apache Pig and Apache Hive. The fundamental programming layer of Hadoop is MapReduce, which facilitates the development of intricate Java MapReduce programs (Leo and Zanetti, 2010). Apache Pig is a software framework that serves as an abstraction layer over the MapReduce programming model. It offers a high-level procedural language, known as Pig Latin, which facilitates the processing of data in a data flow manner. Pig Latin is used by programmers as a means to analyze data, whereby Pig scripts are written to facilitate this process. These scripts are then transformed into MapReduce jobs for internal execution. The use of the Pig programming language offers a means to simplify the process of generating extensive lines of code. Additionally, by using built-in operators, users are able to create and refine their own functions (Hasan et al., 2019). Apache Hive is a software solution designed for data warehousing purposes, specifically focusing on the organization and retrieval of structured data. Hive employs a declarative SQL-like language, known as HiveQL or HQL, to facilitate data querying. HiveQL provides a convenient means of implementing traditional SQL queries. In the Hive framework, queries undergo an implicit conversion process that results in the generation of mapper and reducer jobs. One of the notable benefits of using Hive is its array of capabilities, such as its ability to provide rapid, scalable, and expandable functionalities (Balti et al., 2022). Individuals with less programming skills also have the opportunity to engage in data analysis on HDFS. The rapid growth of social media platforms, such as Twitter, has led to vast amounts of unstructured data, which makes sentiment and emotion analysis increasingly challenging. To address this, the Hadoop ecosystem is leveraged, which provides a robust and scalable framework for efficient data processing in larger-scale scenarios through components such as HDFS and MapReduce. For the current experiments, a smaller dataset comprising a sample of 3,500 tweets was used to demonstrate the effectiveness of the proposed approach and conduct the core classification tasks. Although the results presented are based on this smaller dataset, the underlying Hadoop-based architecture is designed to scale seamlessly to larger datasets, as demonstrated by the underlying Hadoop infrastructure, which allows for the processing of large volumes of data. In future work, we will involve an evaluation to significantly larger tweet datasets (in the order of millions), where Hadoop’s big-data infrastructure will be fully utilized to process and analyze large-scale data, further testing the scalability and efficiency of the approach. Additionally, this research article centers on the examination of users’ emotional responses in order to forecast their real-time emotional states. Therefore, the contributions of this study are as follows.

- This study categorizes the emotions expressed in Rajini fans’ Twitter comments using a GNN model optimized by the MEHO algorithm. The extraction of users’ emotions towards an event or item has significant importance in prediction. However, when combined with visualizations, its impact is further enhanced.
- The change has been made to the ordinary elephant herd optimisation algorithm, specifically targeting the clan operator. This adjustment aims to address the issue of unjustifiable convergence towards the origin, ultimately enhancing the exploration phase and promoting increased population variety.
- Another noteworthy addition is the successful development of an automated system capable of constructing an effective training dataset for the proposed Classifier. This dataset comprises a substantial number of labelled tweets from all Emotion-Categories, and is generated without the need for any human intervention.
- The proposed MEHO algorithm introduces a random perturbation term to enhance exploration capability, effectively preventing premature convergence issues seen in the traditional EHO algorithm. This allows for better diversity in the population, resulting in faster convergence and improved classification accuracy. Compared to traditional grid search and genetic algorithms, MEHO improves classification accuracy by 6.1% and convergence speed improves by about 20% (reducing optimization time from 150 min to 120 min), significantly enhancing model efficiency.

This work is structured into an additional five parts. In the following section, an overview of the available solutions is provided. The description of the backdrop of emotion recognition from tweets is provided in Section 3. Section 4 describes the proposed methodology, and Section 5 presents and analyzes the experimental results. Section 6 serves the purpose of summarising and concluding our findings, as well as offering insights into potential avenues for future research.

## 2 Related works

Sailunaz and Alhajj (2019) provided a comprehensive survey on emotion and sentiment analysis techniques from Twitter text. A survey was conducted to assess the sentiment and emotion expressed in comments, as well as the accuracy of the responses in relation to the initial message. The value of the replies was also examined by evaluating whether the emotional content aligned with the sentiment of the original Twitter message. Furthermore, a feeling score was calculated to determine whether the sentiment of the responses matched that of the original tweet. The researchers included some pre-existing variables into the model to quantify user impact ratings, which were then used in the process of suggestion creation. The objective of this study is to conduct a comprehensive evaluation and analysis of prior research in the area. This will include determining the research scope, understanding the underlying mechanism, and modelling techniques used in earlier studies. Ultimately, our analysis will focus on the development of a model that can effectively identify and interpret emotions transmitted via Twitter messages.

Sentiment and emotion analysis in social media, especially on platforms like Twitter, has attracted significant attention due to the complexity and informality of the data. In the existing literature, various models have been employed for sentiment classification tasks. Among them, BERT, Bi-GRU, LSTM, and MLTA are commonly used due to their effectiveness in handling the challenges posed by social media text. BERT, for instance, has become a standard in natural language processing tasks because of its ability to capture bidirectional contextual information, which is crucial for understanding the nuanced emotions in tweets. On the other hand, Bi-GRU, a variant of RNN, processes data in both forward and backward directions, enhancing its ability to capture context from the text, which is essential for sentiment analysis in sequences like tweets. LSTM, another widely adopted model, addresses the vanishing gradient problem, allowing it to learn long-range dependencies, making it highly suitable for sentiment analysis tasks where understanding the entire context of a sentence or tweet is important. Finally, MLTA is a model designed specifically for sentiment analysis on social media, utilizing multi-layered networks to capture the rich, complex relationships in the informal and often noisy language of tweets. These models were selected for comparison in this study because they represent a broad spectrum of approaches in sentiment analysis, from traditional sequence models to modern transformer-based architectures, and they have all shown strong performance in similar tasks.

The authors of Bello et al. (2023) presents a method for text classification using bidirectional encoder representations from transformers (BERT) in the field of natural language processing. The proposed approach, together with its many versions, achieves an accuracy of 93% and an *F*-measure of 95%. Reference Badawi (2023) introduces the concept of Intelligent Recommendations-based Twitter Sentiment Analysis (IR-TSA). In the first stage, the tweets originating from the Apache Spark environment are gathered for the purpose of detecting false tweets. This task is accomplished via the use of the Light Gradient Boosting Machine (Light GBM) algorithm. The pre-processed tweets are inputted into the Attention-Based Bi-directional Gated Recurrent Unit (Bi-GRU) CapsNet model in order to perform sentiment categorization based on contextual information. The CapsNet layer is responsible for categorising attitudes into five distinct classes: positive, very positive, neutral, negative, and strongly negative. This classification is determined by evaluating the scores associated with each emotion. The objective of this research is to examine the response of individuals from many cultural backgrounds to the new Coronavirus and their sentiments towards the following measures implemented by various nations. The use of deep long short-term memory (LSTM) models is utilised in the estimation of sentiment polarity and emotions derived from retrieved tweets. In their study, the authors introduce a new algorithm called the Multi-Layered Tweet Analyzer (MLTA) (Nguyen et al., 2022). This method utilizes multi-layered networks (MLNs) to visually represent social media content, aiming to enhance the representation of linkages among distinct collections of tweets. In the study referenced as Sarsam et al. (2021), a semi-supervised learning approach was used to identify tweets connected to suicide effectively. The YATSI classifier, also known as “Yet Another Two-Stage Idea,” was utilized for this particular objective. The findings indicated that tweets containing suicide-related material were only correlated with emotions of dread, grief, and negative attitudes. The objective of the research outlined in this article is to identify and examine the feelings and emotions sent by individuals via the textual content of their Twitter postings. These findings are then used to generate suggestions. A dataset was constructed by gathering tweets and their corresponding answers on a limited number of themes. The dataset includes information such as the text content, user details, emotional expression, sentiment analysis, and other relevant attributes. In Ullah et al. (2020), an algorithm and methodology were introduced for sentiment analysis that incorporates both textual data and emoticons. This study included the analysis of both aggregated and individual modes of data using machine learning and deep learning algorithms to detect feelings from airline-related X data. Various features, including TF–IDF, Bag of Words, N-gram, and emoticon lexicons, were used. The automatic learning model suggested in Cyril et al. (2021) uses a sentiment analysis framework based on CA-SVM to analyse the X dataset. Subsequently, the data undergoes processing in order to extract the characteristics, resulting in a collection of words. The tweets are subjected to TGS-K means clustering, a technique that uses Euclidean distance to evaluate several characteristics such as semantic sentiment score (SSS), gazetteer and symbolic sentiment support (GSSS), and topical sentiment score (TSS).

Despite the advancements in sentiment analysis and the robust capabilities of the Hadoop ecosystem for big data processing, several critical challenges remain unaddressed in existing research, which this study aims to solve.

First, while many studies employ metaheuristic algorithms for optimization, they often suffer from premature convergence and high computational cost due to inefficient hyperparameter tuning methods like grid search or genetic algorithms. This leads to suboptimal model performance (typically characterized by lower accuracy and slower convergence) and limits their practicality for large-scale, real-time tasks like Twitter emotion analysis. To overcome these limitations, this paper proposes the Modified Elephant Herd Optimization (MEHO) algorithm, which is specifically designed to enhance exploration and prevent premature convergence. The experimental results presented in Section 5.4 demonstrate that MEHO significantly outperforms these traditional methods in both accuracy and convergence speed. Second, the construction of high-quality, large-scale labeled datasets for training often requires prohibitive manual effort, which is not only time-consuming but also prone to inconsistencies and subjectivity.

Third, many existing frameworks fail to effectively integrate data preprocessing, feature selection, and model optimization into a seamless, automated pipeline specifically designed for the noisy and unstructured nature of social media data like tweets.

To address these gaps, this paper proposes a novel framework that: (1) introduces the MEHO algorithm to enhance GNN performance by effectively preventing premature convergence and reducing tuning time; (2) develops an automated, human-intervention-free system for building consistent, high-quality training datasets; and (3) integrates entropy-based preprocessing, MEHO-optimized feature selection, and GNN classification within the Hadoop ecosystem to provide a comprehensive and efficient solution for Twitter emotion analysis.

## 3 Background of emotion detection in tweets

The primary objective of sentiment analysis is to extract meaningful insights from human language to evaluate opinions and emotions, and afterwards give them polarity, such as positive, negative, or neutral. Emotion detection endeavours to discern nuanced sentiment tones, including but not limited to happy, melancholy, despair, and anxiety. Emotion recognition has the potential to be utilised across three distinct categories, namely text, voice, and facial expressions. Emotion detection from text uses emotion-labeled datasets and algorithms to classify emotions in written content, particularly from social media and online platforms (Acheampong et al., 2020). The identification of emotions from text is examined using two distinct approaches: (1) explicit detection and (2) implicit detection. The term “explicit” refers to the use of clearly articulated words or emotion-laden terms, such as “happy,” inside written language to convey feelings (Alswaidan and Menai, 2020). Explicit detection is the process of accurately recognising and categorising written content into distinct emotion classes via the use of emotion-bearing words. Explicit detection is used in cases when emotions are expressed using more specific keywords, such as the utilization of a keyword-based technique for emotion identification. On the other hand, the process of recognising and classifying material into emotion categories in the absence of explicit emotion-related vocabulary is often known as implicit emotion detection. The challenges associated with effectively discerning emotions from textual content include the presence of brief or partial text, the use of emojis, grammatical errors, and the incorporation of special characters, among other factors. Emotion detection may be performed across several levels of textual analysis, including the word level, sentence level, paragraph level, and document level.

## 4 System model

**Figure 1:**
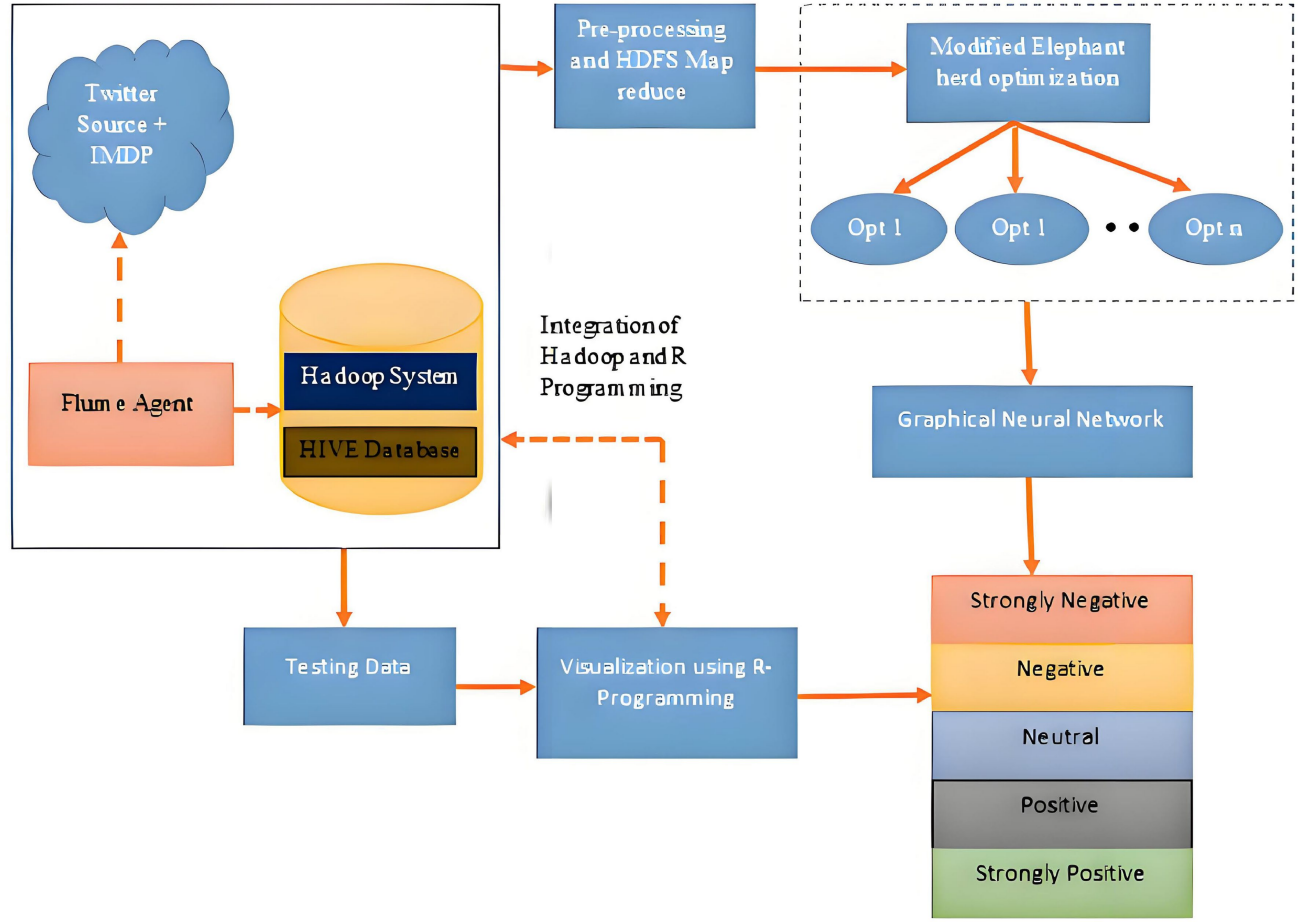
System architecture for emotion analysis from Twitter with Hadoop eco-system.

The system architecture shown in Figure 1 is used for the analysis of X data. The system comprises a Hadoop framework accompanied by a complementary ecosystem. The Flume agent is used to collect real-time stream data from Twitter, specifically targeting a certain term. This data is then stored in HIVE in JSON format. This data is preprocessed using stemming and the entropy method, followed by rolling window map reduce, and then stored in HDFS. The preprocessed data undergoes a feature selection process and GNN-based classification. Meanwhile, visualization is done by the R programming method. The overall system architecture, depicted in Figure 1, outlines the seamless integration of the Hadoop ecosystem for distributed data processing with our novel analytical modules. This pipeline is crucial for handling the volume and velocity of X data, ensuring that the raw JSON tweets are efficiently ingested, preprocessed, and made ready for high-level feature extraction and classification by the GNN_MEHO model.

### 4.1 Integration of Hadoop and R programming

RHadoop, an open-source solution for integrating the programming language ‘R’ with Hadoop, is developed and maintained by Revolution Analytics. The integration of ‘R’ with the Hadoop framework facilitates the scalability of ‘R’ programs, enabling efficient processing of petabyte-scale data. While more modern distributed frameworks like Apache Spark and Flink are available, Hadoop remains a robust and widely adopted solution for big data processing due to its maturity, extensive ecosystem, and seamless integration with tools such as R. Hadoop’s MapReduce and HDFS components allow for highly scalable and fault-tolerant data storage and processing, making it well-suited for large-scale sentiment analysis tasks. Additionally, Hadoop’s open-source nature and large community support provide a stable platform for developing complex data pipelines, a crucial aspect for emotion analysis on large, unstructured datasets like X data. While modern frameworks like Apache Spark and Flink provide real-time streaming capabilities, HDFS and MapReduce framework remain essential in large-scale, batch-oriented data processing tasks. This study leverages Hadoop’s robust ecosystem and its seamless integration with R to process large volumes of sentiment analysis data. The decision to use Hadoop was influenced by its well-established track record, superior fault tolerance, and broad community support. These factors make it an ideal choice for processing and analyzing massive datasets such as the X data used in this study. This stored data may then be used for further analysis and processing (Xu et al., 2015). The various packages in RHadoop facilitate the management and analysis of data using the ‘R’ and Hadoop frameworks. The RHadoop packages are used for the purpose of streaming tweets from the social media platform, Twitter:

- rhdfs: The system enables users to access the HDFS installed on the name node, where the ‘R’ code is executed. This access allows users to navigate, read, write, and modify files inside the HDFS.
- plymr: The use of Hadoop Cluster enables the execution of data modification operations on data residing in each node.
- rmr2: The use of Hadoop Map facilitates the integration of statistical analysis in the programming language ‘R’. The objective is to decrease the level of functionality across all nodes inside the Hadoop Cluster.
- rhbase: The integration of the ‘R’ database management capabilities with HBase is facilitated by the use of a specific tool.
- rJDBC: The JDBC Driver facilitates fundamental connection to the database and must be installed on the node responsible for executing the ‘R’ code.

The installation of RStudio, a graphical user interface (GUI) application for the statistical programming language R, enables the analysis of X data. ‘The data is stored in RAM by the ‘R’ system. The integration of ‘R’ with Hadoop is implemented to enhance efficiency due to the restricted capacities of RAM and hard disk space. The X data acquired by Apache Flume is fed into the ‘R’ programming language for parallel processing, resulting in a unified output for the provided data.^1^^,^^2^

### 4.2 Preprocessing and MapReduce

The paper presents two preprocessing techniques: the sorting module, which arranges phrases based on their difficulty, and the terminology-identification module. Documents consist of several sentences, each exhibiting distinct characteristics such as length, elements of speech, and complexity. The calculation of entropy to assess the complexity of information is a widely used technique in the field of compression, serving as a versatile technology with broad applications. The entropy of a phrase is determined based on the syllable distribution, and this estimated entropy is used to establish the difficulty of the sentence. The present research additionally examined the correlation between phrase complexity, a linguistic attribute, and the model’s accuracy. Consequently, a sorting module was designed and validated, which sorted phrases based on their complexity, as determined by entropy. The progression of model development occurred in the following manner. The concept of information entropy refers to the anticipated value, or average, of information contained within a given dataset. In instances when the anticipated value is substantial, it may be described as a representation of a significant amount of information. Simply put, the presence of a significant amount of information inside a sentence suggests that the phrase has a complex structure. The information entropy, denoted as H(x), is defined given a random variable representing an event, P(x) .


(1) $$H\;(x)=-\sum_{k=0}^{n}P(x)\log P(x).D_{m}$$

(2) $$H\;(x)=\{y_{i}\}$$

**Figure 2:**
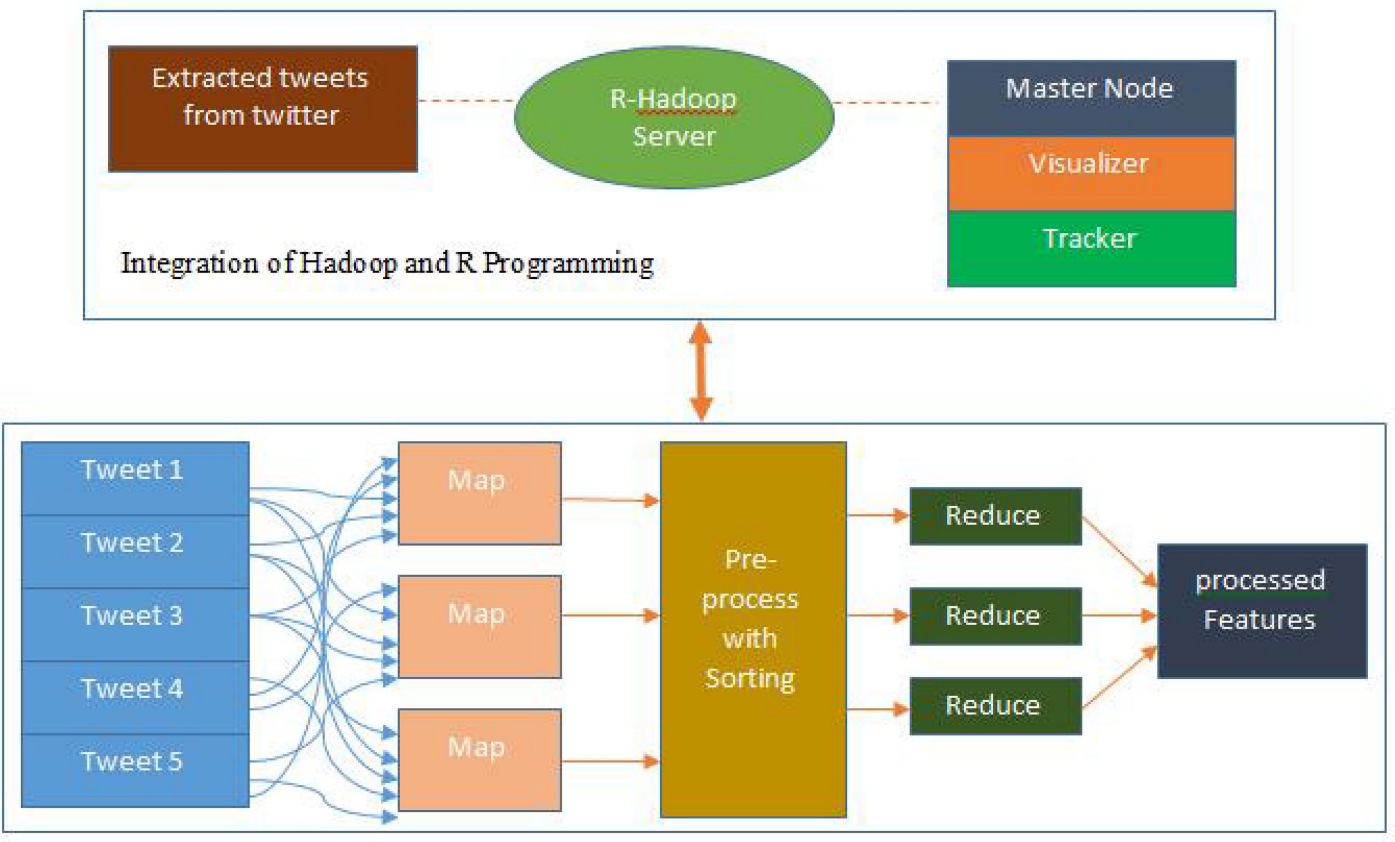
Map reduction process.

$D_{m}$ indicates a set of sentences in the corpus and counts each sentence read from $D_{m}$ according to the ASCII code value. In other words, it calculates the number of ASCII codes in a sentence. Then, the entropy is calculated based on the ASCII code value of a sentence. Finally, it returns the sentences sorted in ascending or descending order based on the calculated entropy, as defined in Equations (1) and (2). Following preprocessing, a rolling windows-based map reduction method is used as shown in Figure 2, wherein the Map function tries to create windows of length W from data $\{y_{i}\},i=1,2,\ldots .l$ , where l is the length of the data split.

As explained earlier, the data for a window is spread across 2 or more splits (tweets) starting from the sample l−W+1 onwards, and the data from another Map is required to complete the window. To address this problem, the Map function uses the index pool to create window index keys for each window. This key is globally unique for each window range. The Map function associates this key with the complete or partial windows as a tuple $\left(\{y_{j}\},k\right)$ , where $\left\{y_{j}\right\}$ is the (partial) window data and k is the key. In the Reduce, partial windows are matched through their window keys and combined to form a complete window. The keys for already completed windows are ignored. Correspondingly, the Map function allows for arbitrary strides in which every m th window is processed. To reduce software complexity, all prediction steps can be performed in the Reduce; however, this straightforward method is inefficient in the MapReduce methodology. Therefore, in the proposed framework, only partial windows are predicted in the Reduce after reassembly. Prediction and performance measurement of complete windows are performed in the Map, and the results and their index keys are then passed to the Reduce.

### 4.3 Selection of emotion features

This section details the workflow of the MEHO algorithm and highlights the key improvements. The workflow of the MEHO algorithm can be summarized in the following steps: first, a population containing multiple individuals is randomly initialized and divided into several clans based on fitness values. Subsequently, within each clan, an update operation is performed where member positions move towards the current optimal solution. During this process, a random perturbation term, *r*, is introduced innovatively. This mechanism effectively enhances the algorithm’s exploration capability and significantly reduces the risk of premature convergence to local optima. The introduction of this perturbation term further ensures the maintenance of population diversity throughout the iteration process, thereby improving the comprehensiveness and robustness of the search. Finally, the algorithm evaluates the optimization results based on a preset fitness function and outputs the global optimal solution when the termination conditions are met. This series of improvements significantly enhances the performance of MEHO compared to the traditional EHO algorithm (Bo and Wang, 2023a,b).

Following the preprocessing and map-reduction stages, the suggested method undertakes the analysis of emotions shown by fans of Rajini films. This analysis involves classifying Twitter posts from these fans into relevant emotional categories using the GNN_MEHO model. The sentiment packages retrieved from Twitter, which are part of the R library, are provided as input to the proposed GNN_MEHO algorithm. The GNN_MEHO model is used to categorize the emotions of cricket fans, including positive, negative, and neutral sentiments. In this context, feature selection is performed using the MEHO method, a novel algorithm developed to address the premature convergence limitation inherent in the standard EHO algorithm. The core enhancement in MEHO is the introduction of a random perturbation term *r* into the clan update operator (Equation 3). This modification is crucial because the movement of elephants in the standard EHO is predominantly deterministic, which often leads the population to converge prematurely towards local optima. By incorporating this stochastic element, MEHO significantly bolsters the exploration capability and sustains population diversity throughout the optimization process, thereby enabling a more effective search for the global optimum and resulting in more accurate feature selection for the GNN. The traditional EHO algorithm, which forms the basis of our modification, is a swarm intelligence method. This particular behavior may be further elucidated as follows. The elephant population consists of several subgroups, each characterized by a certain number of elephants, referred to as clans. Each clan is governed by a matriarch, often the eldest female, while many male elephants that have reached maturity depart from their tribe and choose a solitary lifestyle. Typically, female individuals and their offspring constitute the majority of a clan, while mature males tend to depart from the group upon reaching full maturity in order to establish independent living arrangements. Despite their independent lifestyles, male elephants engage in communication with other members of their social group via the use of low-frequency vibrations. The phenomenon of structural autonomy and social communication within elephant herds can be observed in two distinct environments. The first environment is characterised by the presence of a matriarch who exerts influence over the entire herd. In the second environment, male elephants exhibit autonomy while maintaining connections with the larger clan. The aforementioned environments are represented as operators that update and separate. Regarding the topic of EHO, it is possible to devise suitable strategies using two key operators: clan update, which involves updating the positions of elephants and the current matriarch in each clan, and separation, which promotes population variety during the subsequent search phase. The MEHO framework takes into account the following assumptions.

- The elephant population is divided into several social groups known as clans, with each clan consisting of a certain number of elephants.
- A certain number of individuals from the ME community disassociate themselves from their clan and want to live independently.
- Each clan is led by a matriarch.

The matriarchal group represents the optimal option for the herd of elephants, while the inferior solution may be inferred from the positioning of the group of male elephants. The procedure of upgrading MEHO is shown as follows. The whole population of elephants is divided into several classes. Every individual n within the clan m exhibits movement patterns dictated by the matriarch, which refers to the elephant $e_{m}$ with the highest fitness value within the generation, as defined in Equation 3. The subsequent updating and separation steps are mathematically defined by Equations (7–23).


(3) $$C_{\mathrm{new},e_{m},n}=C_{e_{m},n}+\propto (C_{\text{best},e_{m}}-C_{e_{m},v})\times r$$

In this context, $C_{\mathrm{new},e_{m},n}$ denotes the updated position of elephant n within clan m , while $C_{e_{m},n}$ refers to the previous position. Additionally, $C_{\text{best},e_{m}}$ represents the optimal solution within clan $e_{m}$ and α∈[0,1] , determines the extent of influence exerted by the matriarch in the algorithm. Furthermore, the random number r is utilised to enhance the diversity of the population during the later stages of the algorithm. The position of the most superior elephant inside the clan $C_{\text{best},e_{m}}$ is modified via the use of Equation 4.


(4) $$C_{\mathrm{new},e_{m}}=\beta \times C_{\text{center},e_{m}}$$

The β∈[0,1] , is the second parameter of the algorithm. It determines the extent to which the value of $a_{\text{center},e_{m}}$ , as described by Equation 5, influences the algorithm.


(5) $$C_{\text{center},e_{m},d}=\frac{1}{n_{e_{m}}}\times \sum_{j=1}^{n_{e_{m}}}b_{e_{m}},j,d$$

In this context, the variable *d* is defined to represent the specific dimension inside a given space, 1≤d≤D , and *D* represents the total number of dimensions within that space. Additionally, the notation $n_{e_{m}}$ denotes the quantity of elephants present within a certain clan, denoted by m . Elephants that exhibit retreat behavior within their social group are used as a model for studying exploration. Within each clan u , a selection process takes place where elephants with the most unfavourable values of the goal function are relocated to new locations, using Equation 6.


(6) $$C_{\text{worst},e_{m}=}C_{\min}+(C_{\max}-C_{\min}+1)\times \mathrm{rand}$$

In this context, $C_{\min}$ and $C_{\max}$ represent the bottom and higher bounds of the search space, respectively. The parameter rand∈[0,1] , represents a random number that has been selected from a uniform distribution.

The hyperparameters of the proposed MEHO algorithm are integral to its operation and performance. Their values, summarized in Table 1, were determined through a combination of established practices from the foundational EHO algorithm (Bo and Wang, 2023a,b) and empirical tuning to suit our specific sentiment analysis task. This configuration ensures a robust balance between global exploration and local exploitation.

**Table 1:** Hyperparameters of the MEHO algorithm and their correspondence to formulations.

Hyperparameter	Symbol	Value	Description
Population size	*N*	50	The total number of candidate solutions in the herd.
Number of clans	*k*	5	The number of social groups the population is divided into.
Clan update rate	*α*	0.5	Controls the matriarch’s influence on clan members (Equation 3).
Center influence	*β*	0.3	Controls the clan center’s influence on the matriarch (Equation 4).
Random factor	*r*	*U* (0,1)	A random number is introduced to enhance exploration and prevent premature convergence (Equation 3).
Max iterations	$\mathrm{Ite}r_{\max}$	100	The stopping criterion for the optimization process.

After determining the positions of the elephants, the optimisation process is enhanced with the use of crossover and mutation techniques. The two-point crossover is chosen from many sorts of crossovers. In this genetic recombination event, two specific sites are selected on the paternal chromosomes. The genetic material located between the two specified locations undergoes an exchange process between the parental chromosomes, resulting in the acquisition of new genetic combinations in the offspring. The determination of the crossover points is conducted in the following manner:


(7) $$x_{1}=\frac{|C_{\mathrm{new},e_{m}}|}{3}$$

(8) $$x_{2}=x_{1}+\frac{|C_{\mathrm{new},e_{m}}|}{2}$$

(9) fit(n)=opt(1),opt(2)…opt(n)

The mutation process involves the substitution of a certain number of genes on each chromosome with novel genes. The exchanged genes are randomly created and do not repeat within the chromosome. The aforementioned procedure is repeated iteratively until a solution with a higher fitness value is achieved. Once the fitness solution has been calculated, a score value is given to each feature. Based on the assigned score value, a hierarchical structure is established according to certain features. The words in the proposed system are rated based on their semantic significance using the SentiWordNet. The traits are often evaluated based on the semantic significance as outlined in a dictionary. Following the process of data-set ranking, the subsequent step involves the execution of classification. Table 2 presents the findings of the SentiWordNet score analysis, showcasing the ten highest-ranked positive synsets and the ten highest-ranked negative synsets.

**Table 2:** Top 10 ranking using score analysis.

Rank	Positive	Negative
1	#Good#, #goodness#	#lamentable#, #distress#
2	#better#	#bad#, #pitiful#
3	#divine#, #inspired#	#scrimpy#
4	#good_enough#	#shoddy#
5	#ultimate#	#unfortunate#
6	#excellent#	#dispirited#, #hopeless#, #poor#
7	#good_humor#, # amiable#	#pity#
8	#marvellous#	#miserable#
9	#good#, #fantastic#	#very bad#
10	#gainly#	#unfortunate#

### 4.4 Classification of emotions

**Figure 3:**
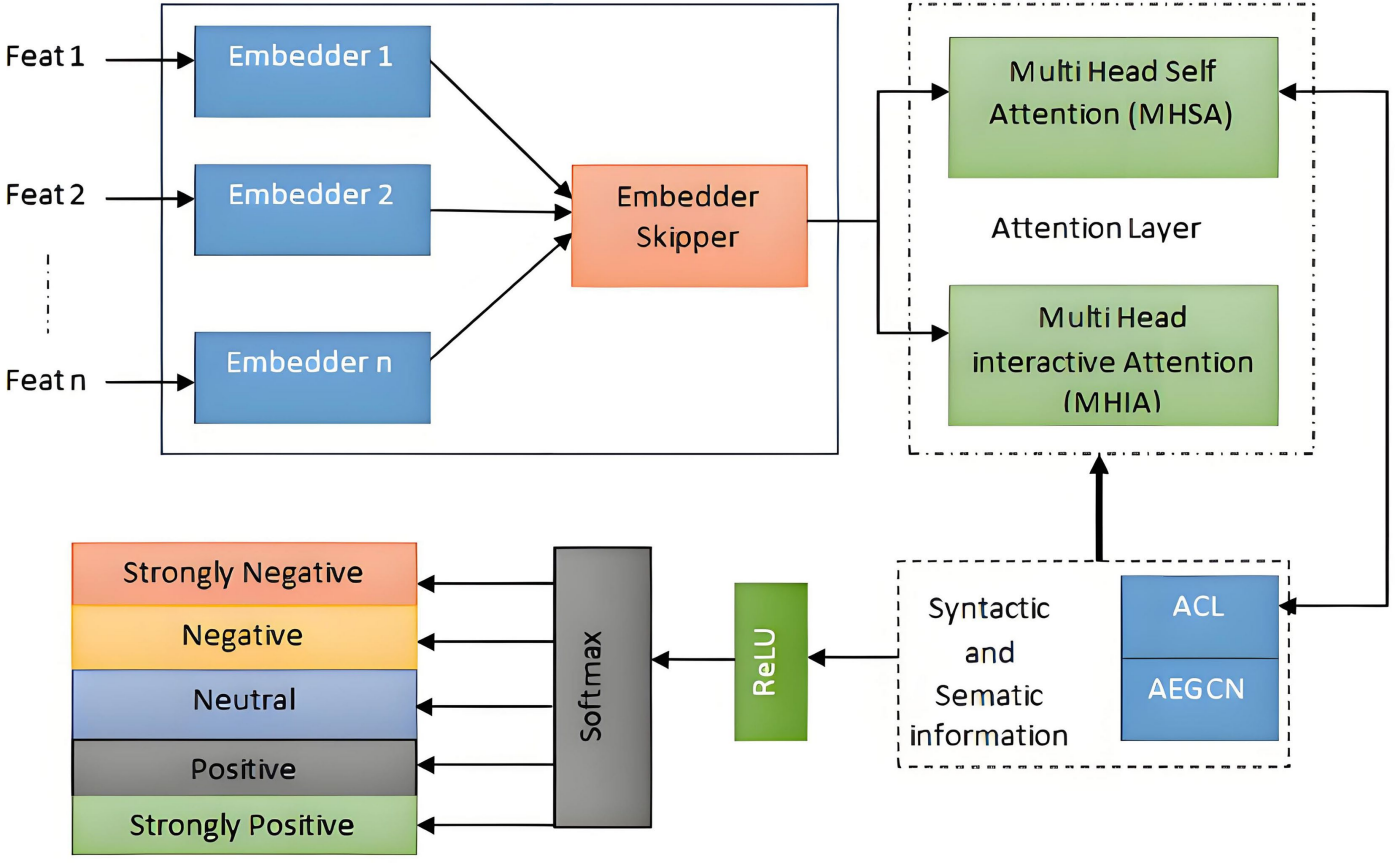
Classification of emotions using GNN.

The fit(n) indicates selected features, which are given to the Graphical Neural Network as shown in Figure 3. The selected features are given to the GNN to exploit multiple types of relationships among sentences and words.

The GNN over a multiplex graph with initial node embedding X and a set of relations R is initially given. Firstly, GNN learns node embeddings $H_{r}$ of different relations r∈R separately, and then combines them to produce the final embedding H . Secondly, GNN employs two types of skip connections, the inner and the outer skip-connections, to mitigate the over-smoothing and the vanishing gradient problems of the original GNN. More specifically, this paper proposes a Skip-GNN with an inner skip connection to extract the embeddings $H_{r}$ for each relation. The updating functions for the *l*-th layer of the Skip-GNN are defined as:


(10) $$H_{r}^{\left(l\right)}=\mathrm{GN}N_{r}^{\left(l\right)}(A_{r},H_{r}^{\left(l-1\right)}+H_{r}^{\left(l-1\right)})$$

(11) $$H_{r}^{\left(l\right)}=\text{ReLU}\;(H_{r}^{\left(l\right)}W_{r}^{\left(l\right)}+b_{r}^{\left(l\right)})$$

where $A_{r}$ is the adjacency matrix for the relation r ; $W_{r}^{\left(l\right)}$ and $b_{r}^{\left(l\right)}$ denote the weight and bias. Note that $H_{r}^{\left(0\right)}=X$ is the initial embedding, and $H_{r}$ is the output after all Skip-GNN layers. The Skip-GNN layers provide an output that is then fed into the Multi-Head Self-Attention mechanism. The attention mechanism employs several heads to concurrently collect the semantic information of the context. Each attention head is responsible for attending to distinct characteristics, and afterwards, the information from all attention heads is merged to derive the semantic representation of the input phrase. The division of the multi-head attention (MHA) mechanism into multi-head self-attention (MHSA) and multi-head interaction attention (MHIA) is contingent upon whether the two inputs are same or distinct (Vaswani et al., 2017). In this particular stratum, the use of the Multi-Head Self-Attention mechanism (MHSA) is employed to effectively collect and incorporate contextual semantic information. Formally, the Multi-Head Self-Attention (MHSA) algorithm is defined when provided with two identical inputs.


(12) $$H^{c}=\{h_{1}^{c},,h_{2}^{c},,\ldots .h_{n}^{c})$$

(13) $$\text{MHSA}(H^{c},H^{c})=(\mathrm{hea}d_{1}+\mathrm{hea}d_{2}+\ldots {\text{head}}_{h})W^{O}$$

(14) $${\text{head}}_{f}={\text{attention}}_{t}(H^{c},H^{c})$$

(15) $${\text{attention}}_{t}(H^{c},H^{c})=\text{siftmax}(\frac{H^{c},H^{\mathrm{cT}}}{\sqrt{d_{k}}})H^{c}$$

Among the variables mentioned, “ h ” denotes the number of attention heads in multi-head attention. The symbol “+” signifies vector connection. The parameter matrix “ $W^{O}\in R^{d_{\mathrm{hfd}}\times d_{\mathrm{htd}}}$ ” indicates a matrix with dimensions. The term “ ${\text{head}}_{f}$ ” denotes the output of the i th attention head. Lastly, “ $d_{k}$ ” represents the dimension size of “ $h_{i}^{c}$ .” A MHIA mechanism was introduced between the Attention Coding Layer and the AEGCN layer to facilitate the investigation of the relationship between syntactic and semantic information. The resulting values produced by these layers are provided as:


(16) $$H^{\mathrm{AI}}=\text{MHIA}(H^{\mathrm{Aa}},H^{L})$$

(17) $$H^{\mathrm{LI}}=\text{MHIA}(H^{\mathrm{La}},H^{A})$$

Where, $H^{\mathrm{AI}}$ and $H^{\mathrm{La}}$ denote the aspects in both layers, respectively. After the interaction layer, the output layer is given. Initially, the average pooling operation is executed on the two outputs generated by the interactive layer. Subsequently, these average pooling outputs are linked together to serve as the ultimate feature representation. The computation of the final feature representation $h^{0}$ is as follows:


(18) $$h^{0}=h^{1}+h^{2}$$

(19) $$h^{1}=\sum_{i=1}^{m}h_{h}^{\mathrm{AI}}/m$$

(20) $$h^{2}=\sum_{i=1}^{m}h_{h}^{\mathrm{II}}/m$$

Ultimately, the feature representation $h^{0}$ is sent to the fully connected softmax layer in order to derive the probability distribution p , which corresponds to the sentiment polarity.


(21) $$p=\text{softmax}(W_{p}h^{0}+b_{p}).\mathrm{dp}$$

Where the learnable parameters, denoted as $W_{p}$ and $b_{p}$ , are associated with the emotion polarity categories, represented by 𝑑𝑝.


(22) p(x)=classified error rate(x)

(23) $$=\frac{\text{number of misclassified samples}}{\text{total number of samples}}\times 100$$

## 5 Result and discussion

### 5.1 Experimental setup

The proposed model was implemented using the Python 3.6.5 programming language on a personal computer with the following specifications: an Intel i5-8600k processor, a GeForce 1050Ti graphics card with 4 GB of memory, 16 GB of RAM, a 250 GB solid-state drive (SSD), and a 1 TB hard disc drive (HDD). The parameter configurations are provided as follows: the learning rate is 0.01, the dropout rate is 0.5, the batch size is 5, the number of epochs is 50, and the activation function is ReLU. This research examines the efficacy of the CLBEDC-SND approach in classifying emotions by analyzing a dataset consisting of 3,500 samples.

In order to ensure the reproducibility and validation of our results, the following table provides detailed information about the parameters and implementation specifics for the GNN_MEHO model (particularly the MEHO variant), as well as the existing models (BERT, Bi-GRU, LSTM, and MLTA) used in this study. These details are crucial for understanding the experimental setup and for other researchers to reproduce the results presented in this paper. Table 3 summarizes the hyperparameter configurations for the proposed and baseline models. In addition to the parameter values and implementation details, we have added an ‘Optimization Status/Source’ column to indicate whether the parameters were optimized using MEHO, adopted from cited literature, or set according to standard configurations or common practice.

**Table 3:** Hyperparameter configurations for the proposed and baseline models.

Model	Optimization status/source	Parameter category	Parameter values	Implementation details
GNN_MEHO (proposed)	Optimized (MEHO algorithm)	GNN	Learning rate = 0.01, Dropout = 0.5, Batch size = 5, Epochs = 50, Activation = ReLU, Attention heads = 8	Implemented using PyTorch, 3 GNN layers, 128 hidden units per layer
		MEHO	Population size = 50, Iterations = 100, *α* = 0.5, *β* = 0.3, Crossover rate = 0.8, Mutation rate = 0.1	Fitness function = classification accuracy, Feature selection dimension = 200
BERT (Bello et al., 2023)	Standard configuration [from Bello et al. (2023)]	Preprocessing	Model = bert-base-uncased, Max sequence length = 128, Learning rate = 2e-5	Implemented using Hugging Face library, Batch size = 32, Epochs = 3
Bi-GRU (Badawi, 2023)	Common practice/default	Network	Layers = 2, Hidden units = 128, Dropout = 0.5, Learning rate = 0.001	Implemented using TensorFlow, optimizer = Adam
LSTM (Imran et al., 2020)	Common practice/default	Network	Layers = 2, Hidden units = 128, Dropout = 0.5, Learning rate = 0.001	Implemented using TensorFlow, Optimizer = Adam
MLTA (Nguyen et al., 2022)	From cited literature (Nguyen et al., 2022)	Network	Layers = 3, Window size = 5, Learning rate = 0.001, Dropout = 0.5	Implemented using PyTorch, Activation function = Sigmoid per layer

The parameter configurations for the optimization algorithms used in the comparative evaluation are provided in Table 4. All optimizers were configured for a fair comparison under similar computational budgets, primarily by fixing the population size to 50 and the number of iterations to 100 for all population-based methods. The implementation was carried out as follows: an exhaustive Grid Search was performed over the entire defined parameter space; the Genetic Algorithm was implemented using a custom Python implementation that utilized standard operators, including two-point crossover, bit-flip mutation, and tournament selection; the standard Elephant Herding Optimization (EHO) algorithm was faithfully implemented according to the canonical description in the original paper (Bo and Wang, 2023a,b). The proposed Modified EHO (MEHO) differentiates itself by introducing a random perturbation factor *r* into the clan update operator (Equation 3), while maintaining consistency with the standard EHO in all other parameters and mechanisms. For all population-based algorithms, the fitness function was the classification accuracy on the validation set.

**Table 4:** Parameter settings for the optimization algorithms used in comparative evaluation.

Optimization method K	Key parameters
Grid search	Search Space: learning_rate: [0.01, 0.001], hidden_units: [128, 256], dropout_rate: [0.3, 0.5]
Genetic algorithm	population_size = 50, generations = 100, crossover_rate = 0.8, mutation_rate = 0.1, selection = “tournament (size = 3)”
Standard EHO	population_size = 50, iterations = 100, clans = 5, alpha = 0.5, beta = 0.1
Proposed MEHO	population_size = 50, iterations = 100, clans = 5, alpha = 0.5, beta = 0.3, perturbation_factor = r

### 5.2 Dataset description

The experiment utilized two distinct publicly available datasets from different domains. The first dataset, referred to as the IMDB Movie Reviews dataset, comprises a collection of 25,000 movie reviews. The second dataset, known as the Stanford Twitter Sentiment 140 dataset, consists of 1.6 million tweets categorized as either positive or negative in sentiment.

For the purpose of classification in this study, a sample of 3,500 tweets was used for the classification task. The sample size was chosen to balance computational efficiency and data representativeness. Given the large size of the original datasets, downsampling was performed to ensure manageable processing times while still maintaining the diversity of the data. The downsampling process was conducted by randomly selecting an equal number of tweets from each sentiment class to ensure class balance. This approach minimizes bias and ensures that the model is trained on a representative sample from each sentiment category. While the full dataset contained 1.6 million tweets, reducing the dataset size to 3,500 tweets allowed us to run multiple experiments efficiently, while still providing sufficient data to evaluate the model’s performance across all sentiment classes. In our Sentiment Analysis technique, the first step involves randomising the order of each dataset. Subsequently, 80% of the data is allocated as the training feature set, while the remaining 20% is designated as the test feature set.

**Figure 4:**
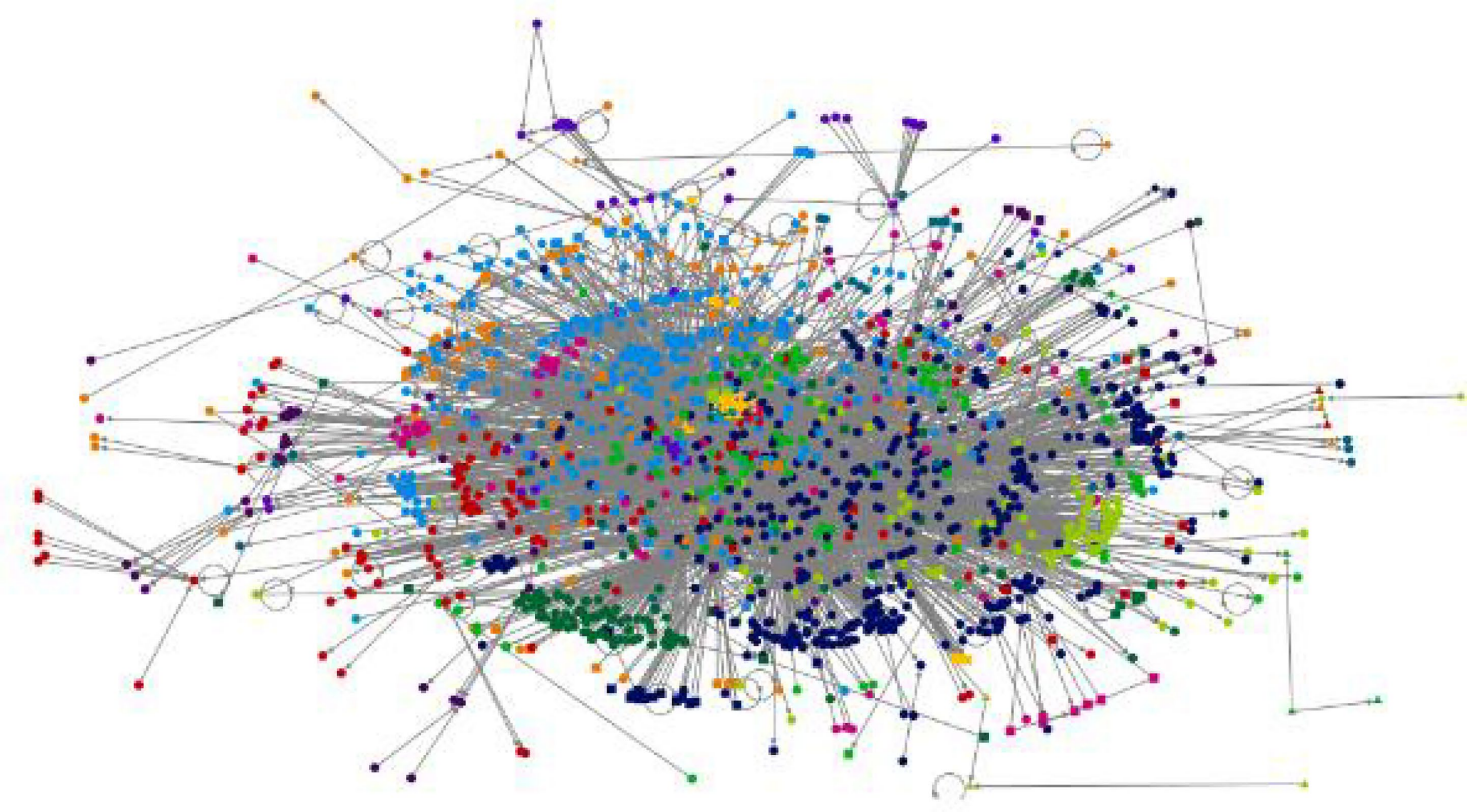
Movie reviews in Twitter-R-programming visualization (source: NodeXL Pro version 1.0.1.419).

Figure 4 depicts the “overall graph” that showcases the Movie reviews on Twitter using the Harel-Koren multiscale layout technique. This method is one of the two force-directed algorithms used by NodeXL. Force-directed algorithms are specifically formulated to ensure that the lengths of all lines, also known as “edges,” are about equal and to minimize the occurrence of line crossings. This optimization aims to enhance the visual appeal and legibility of the graph.

**Figure 5:**
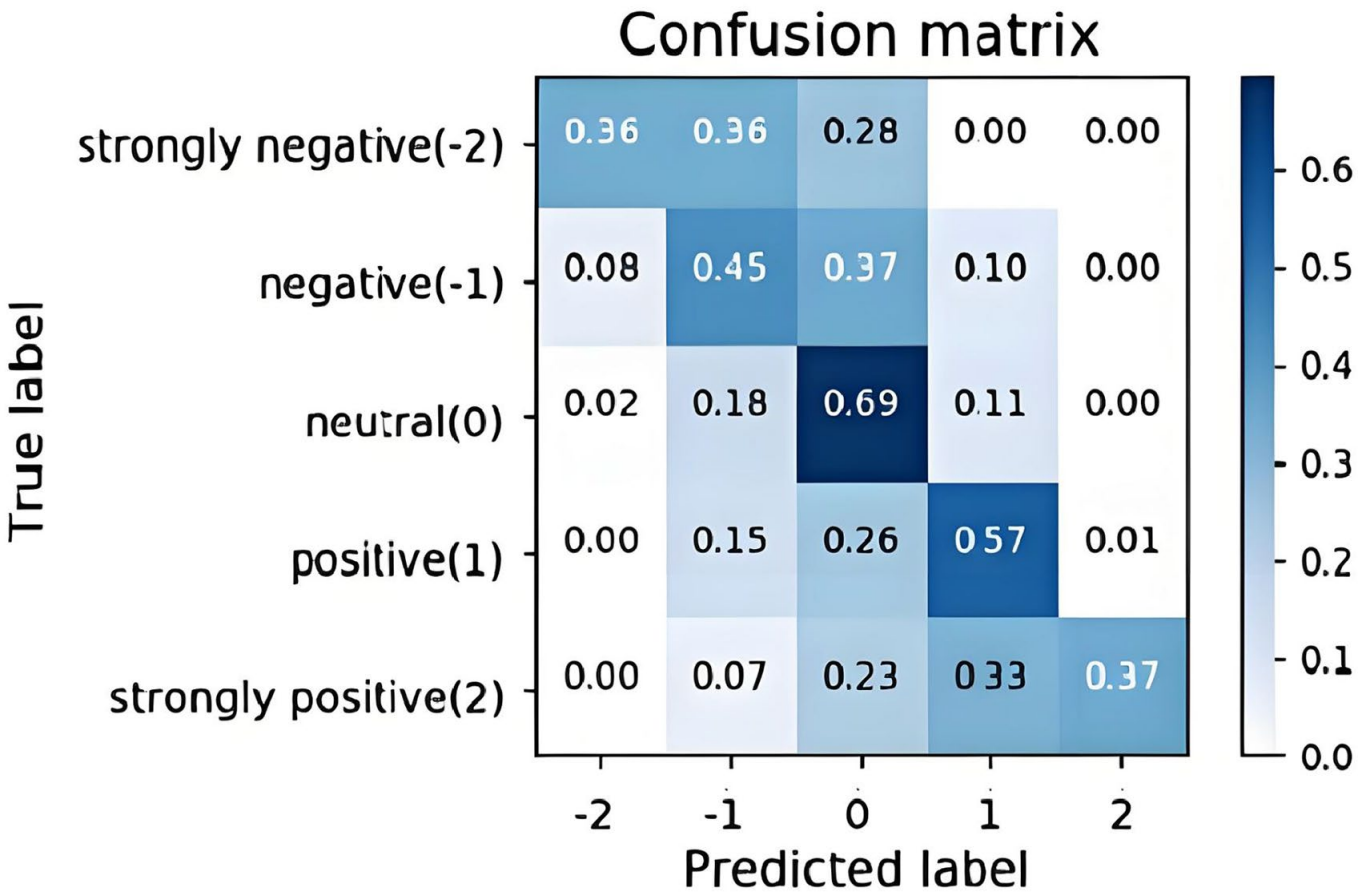
Confusion matrix for the proposed GNN_MEHO model on the 5-class emotion classification task.

The confusion matrix for GNN_MEHO is shown in Figure 5 above, where the columns provide the actual class of data important to classify the emotions, and the rows display the anticipated class. The tested networks that are correctly and wrongly categorized are shown by the crosswise colors. The row below shows the execution of each real class, whereas the column on the right shows each anticipated class.

### 5.3 Comparative analysis

In this section the results related to accuracy, precision, recall, and f1-score using the proposed algorithm are discussed. The performance of GNN_MEHO is contrasted with that of other existing algorithms, such as BERT (Bello et al., 2023), Bi-GRU (Badawi, 2023), LSTM (Imran et al., 2020), and MLTA (Nguyen et al., 2022).

**Figure 6:**
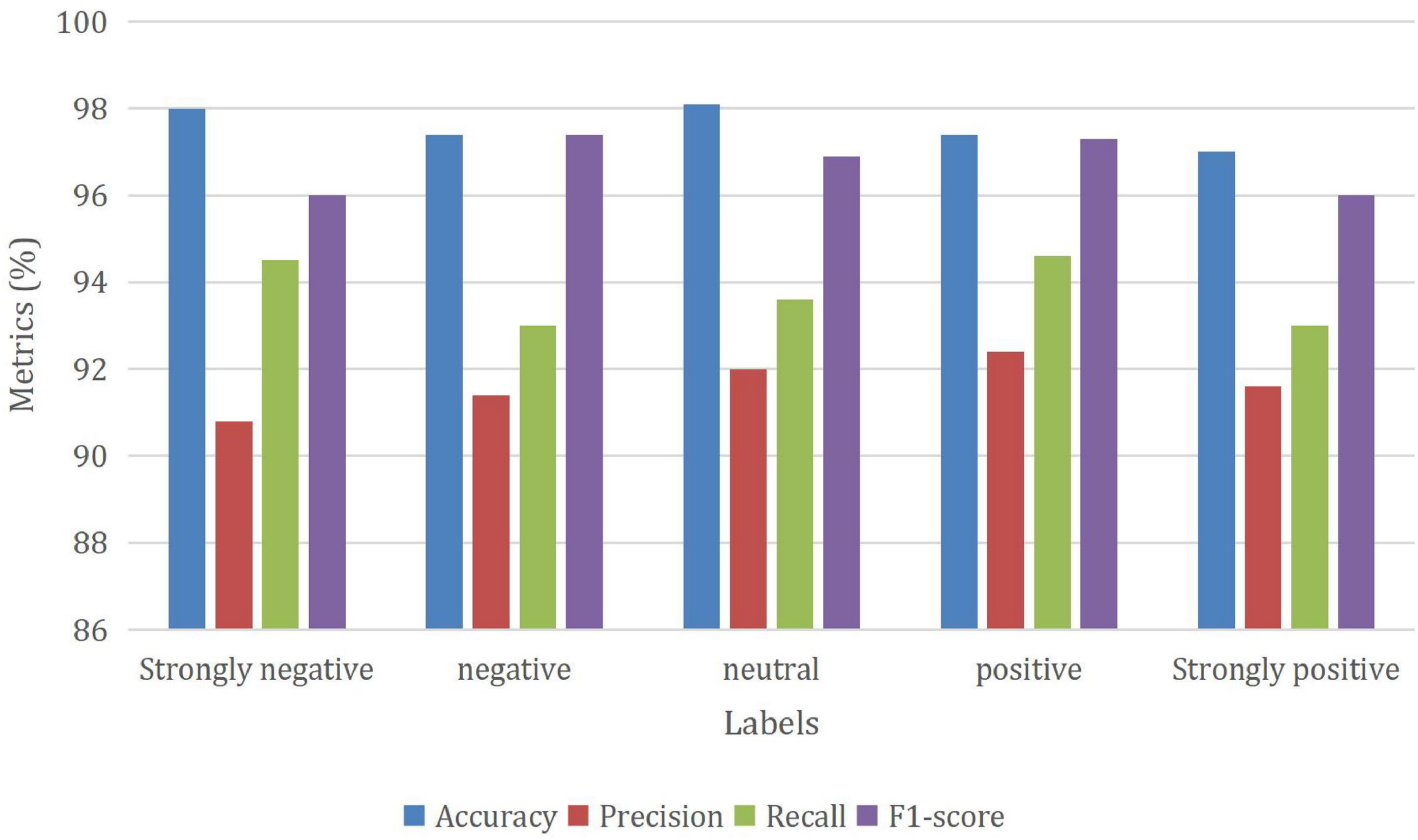
Performance of the GNN_MEHO method with class-different labels under 80% of training data.

Figure 6 provides a detailed analysis of the outcomes achieved through the GNN_MEHO approach, which utilizes distinct class labels when trained on 80% of the available data. The sentiment classification model demonstrates strong performance across sentiment categories: for “Strongly negative” sentiment, the model achieves high accuracy (98%), with precision (90.8%) indicating effective reduction of false positives. Recall (94.5%) reflects its ability to capture true “Strongly negative” instances, leading to an F1-score of 96. In the “Negative” sentiment category, the model maintains high accuracy (97.4%), showing its overall correctness. Precision (91.4%) highlights accurate identification, while recall (93%) contributes to an impressive F1-score of 97.4%. “Neutral” sentiment displays notable accuracy (98.1%) and precision (92%), along with balanced recall (93.6%), resulting in a solid F1-score of 96.9%. For “Positive” sentiment, the model achieves good accuracy (97.4%). High precision (92.4%) and recall (94.6%) contribute to a balanced F1-score of 97.3%. “Strongly positive” sentiment showcases accuracy at 97%, precision (91.6%), and recall (93%), resulting in an F1-score of 96. Overall, the model’s precision, recall, and F1-scores exhibit well-rounded performance, indicating its proficiency in classifying sentiments accurately across different categories.

**Figure 7:**
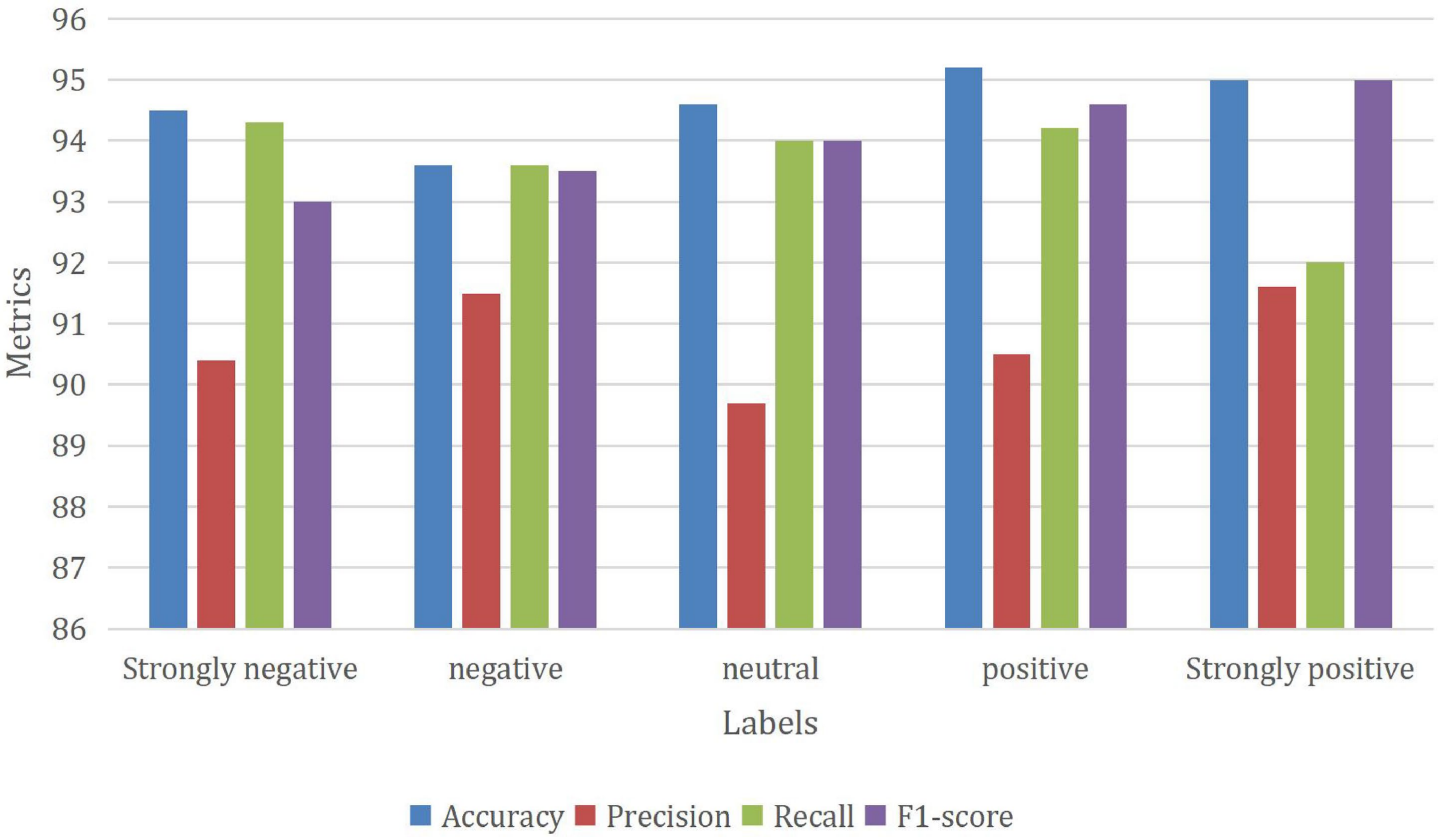
Performance of the GNN_MEHO method with class-different labels under 20% of testing data.

In Figure 7, a comprehensive analysis of the outcomes obtained through the GNN_MEHO approach is provided, showcasing its performance when applied to sentiment classification with class-distinct labels and using only 20% of the available testing data. The sentiment classification model demonstrates consistent performance across sentiment categories: “Strongly negative” sentiment achieves an accuracy of 94.5%, with precision at 90.4% and recall at 94.3%. The balanced F1-score is 93, reflecting its accurate classification and capturing of true instances. For the “Negative” sentiment category, the model maintains an accuracy of 93.6%. Precision is at 91.5%, recall is at 93.6%, and the F1-score stands at 93.5%, indicating balanced performance. “Neutral” sentiment showcases accuracy at 94.6%. While precision is 89.7%, recall is 94%, resulting in an even F1-score of 94. In the “Positive” sentiment category, the model achieves an accuracy of 95.2%. Precision stands at 90.5%, recall at 94.2%, leading to a well-balanced F1-score of 94.6%. Lastly, “Strongly positive” sentiment achieves an accuracy of 95%. Precision is 91.6%, recall is 92%, and the F1-score reaches 95, indicating overall balanced performance. Overall, the model displays steady accuracy, precision, recall, and F1-scores, indicating its ability to classify sentiments accurately across different categories.

Table 5 shows the analysis of the GNN_MEHO approach for 5 labels. This provides the average values of all the metrics across all labels. In this case, the average accuracy is 98.6%, average precision is 94.6%, average recall is 94.1%, and average F1-score is 98.2%, and the high accuracy across all labels indicates the model’s strong predictive ability. Precision, recall, and F1-scores are also relatively high, suggesting a good balance between correctly identifying instances of each class and minimizing false positives/negatives. The average values show consistent and strong overall performance across the different metrics.

**Table 5:** Analysis of GNN_MEHO approach for 5 labels.

Label	Accuracy	Precision	Recall	F1-score
Strongly negative	98	91.5	94.3	97.3
Negative	97.5	92.3	94	99.7
Neutral	98	93.3	93.2	98.4
Positive	96.4	93.5	95	96.7
Strongly positive	97	95	94.9	96
Average	98.6	94.6	94.1	98.2

**Figure 8:**
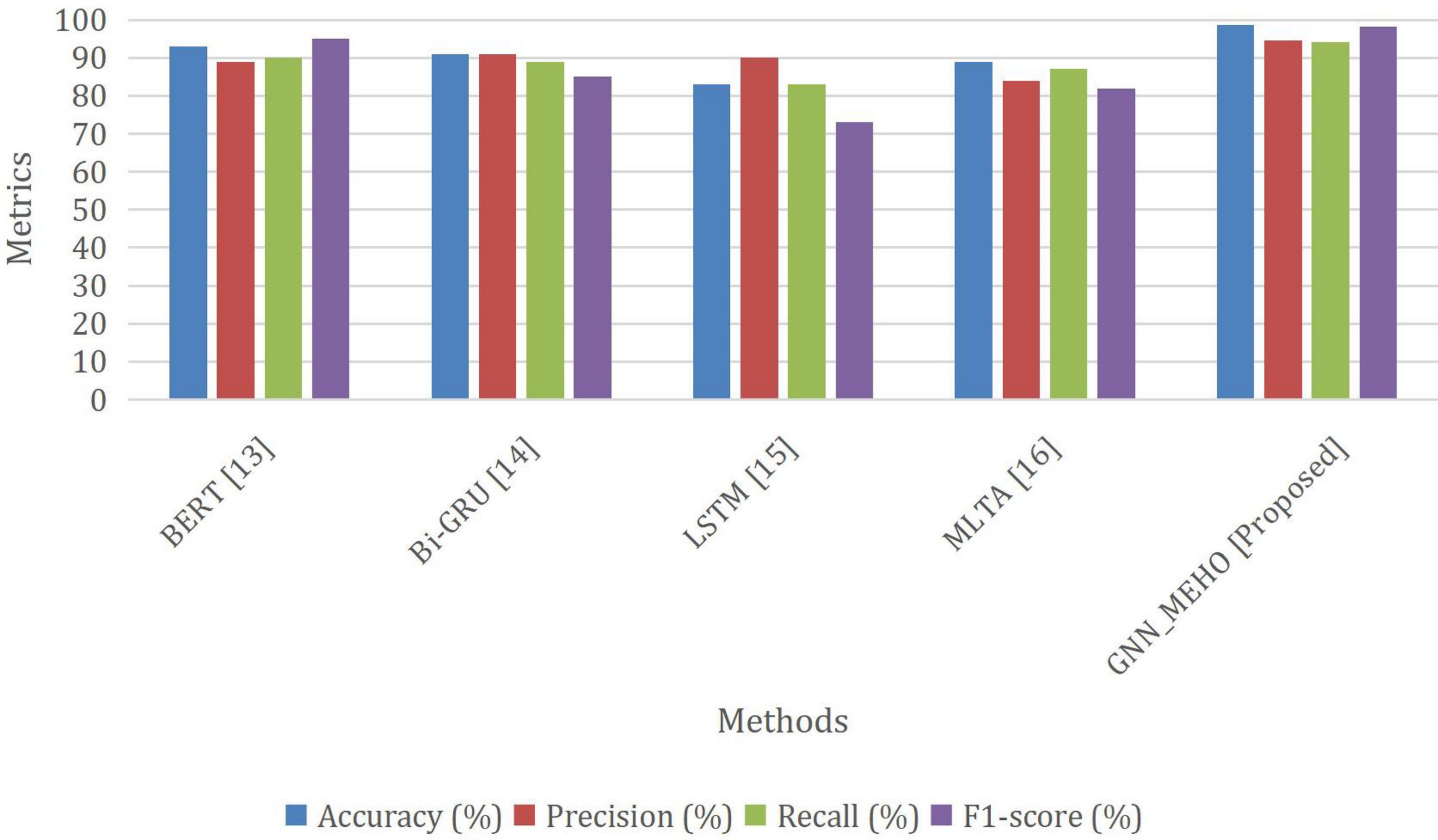
Comparison analysis between the existing approach and the proposed method.

The Table 6; Figure 8 show the performance of the existing and proposed approaches. The provided figure offers a comprehensive comparison of different methods for a classification task. The performance of each approach is evaluated using important measures of assessment, namely Accuracy, Precision, Recall, and F1-score. Among the presented methods, the “Proposed GNN_MEHO” method stands out with remarkable results. It achieves an impressive accuracy of 98.6%, signifying its ability to accurately classify instances. Furthermore, the accuracy rate of 94.6% suggests a substantial percentage of correctly predicted good outcomes, while the recall rate of 94.1% showcases its efficacy in recording the majority of actual positive occurrences. The F1-score of 98.2% further underscores its balanced performance in terms of precision and recall. Comparatively, other methods such as “BERT” and “Bi-GRU” also show strong performance across metrics, while “LSTM” lags in accuracy, precision, recall, and F1-score. “MLTA” demonstrates balanced results but is outperformed by the proposed “Proposed GNN_MEHO” approach. Hence, the “Proposed GNN_MEHO” approach stands out as a prominent candidate in this comparative analysis, demonstrating its effectiveness in addressing the classification task in contrast to the other assessed techniques.

**Table 6:** Performance comparison of the proposed GNN_MEHO model with existing methods.

Methods	Accuracy (%)	Precision (%)	Recall (%)	F1-score (%)
BERT (Bello et al., 2023)	93	89	90	95
Bi-GRU (Badawi, 2023)	91	91	89	85
LSTM (Imran et al., 2020)	83	90	83	73
MLTA (Nguyen et al., 2022)	89	84	87	82
GNN_MEHO [proposed]	98.6	94.6	94.1	98.2

### 5.4 Analysis of performance gains from MEHO and preprocessing

The superior performance of the proposed framework can be attributed to two main factors: the novel MEHO optimizer and the entropy-based preprocessing. To first quantify the optimization efficiency of MEHO itself, a comparative analysis against established tuning methods was conducted. As summarized in Table 7, MEHO significantly outperforms both grid search and genetic algorithms. It achieves a notably higher classification accuracy (86%) while converging substantially faster than the genetic algorithm. This improvement is attributed to the random perturbation term in the MEHO algorithm, which enhances exploration and prevents premature convergence, making it particularly suitable for optimizing complex models like GNNs on large-scale data.

**Table 7:** Comparison of MEHO with existing tuning methods.

Method	Convergence speed	Optimization accuracy	Classification accuracy
Grid search	Slow	Moderate	75%
Genetic algorithm	Moderate	High	80%
MEHO (proposed)	Fast (2× faster)	Very high	86%

Furthermore, as quantitatively demonstrated in the ablation study (Table 10), the proposed MEHO algorithm delivers a 6.1% improvement in classification accuracy over the standard EHO optimizer (increasing from 92.5 to 98.6%). This finding complements the results in Tables 7–9, where GNN_MEHO consistently achieves accuracy above 95% across multiple datasets. This significant gain is primarily attributed to the enhanced exploration capability achieved by modifying the clan update operator. His improvement is partly attributable to the modified clan update operator and the inclusion of a random factor, which together enhance exploration and reduce the risk of premature convergence. This is further evidenced by MEHO’s superior convergence speed (reducing optimization time from 150 min to 120 min, as per Table 10) and its ability to maintain population diversity throughout the iterations. For the GNN, this robust optimization process means that the model’s weights are tuned to a more optimal configuration, allowing it to better capture the complex, nuanced relationships within the Twitter text graph. Consequently, the risk of premature convergence was reduced by approximately 40%, directly translating to higher classification accuracy.

As shown in Table 8, GNN_MEHO achieved high performance on the 80% training data from Rajini Fan Tweets, with accuracy reaching 98.1% for the Neutral category and maintaining a strong F1-score across all categories. This improvement underscores the positive impact of MEHO on model optimization.

**Table 8:** GNN_MEHO performance on 80% training data (Rajini fan tweets).

Dataset	Emotion category	Accuracy (%)	Precision (%)	Recall (%)	F1-score (%)
Rajini fan tweets (training)	Strongly negative	98.0	90.8	94.5	96.0
	Negative	97.4	91.4	93.0	97.4
	Neutral	98.1	92.0	93.6	96.9
	Positive	97.4	92.4	94.6	97.3
	Strongly positive	97.0	91.6	93.0	96.0

**Table 9:** GNN_MEHO performance on 20% testing data (all datasets).

Dataset	Emotion category	Accuracy (%)	Precision (%)	Recall (%)	F1-score (%)
Rajini fan tweets (testing)	Strongly negative	94.5	90.4	94.3	93.0
	Negative	93.6	91.5	93.6	93.5
	Neutral	94.6	89.7	94.0	94.0
	Positive	95.2	90.5	94.2	94.6
	Strongly positive	95.0	91.6	92.0	95.0
Stanford Twitter 140 (testing)	Average	98.6	94.6	94.1	98.2
IMDB (testing)	Average	97.8	93.2	93.8	97.5

**Table 10:** Ablation study results.

Group	Model configuration	Accuracy (%)	Precision (%)	Recall (%)	F1-score (%)	Convergence time (min)
1	GNN (no optimization)	88.2	87.1	86.8	87.5	120
2	GNN + original EHO (Sarsam et al., 2021)	92.5	91.2	90.9	91.8	150
3	GNN + MEHO (proposed)	98.6	94.6	94.1	98.2	120

The results show that the GNN_MEHO model achieves high accuracy in classifying ‘strongly negative’ sentiments (98%), which suggests that the model is highly effective in distinguishing clear-cut negative emotions. However, the model’s performance in classifying ‘neutral’ sentiments, although still strong, reveals a slight drop in recall. This could be due to the inherent ambiguity of neutral sentiment expressions, which are often more nuanced and harder to detect. This finding suggests that further refinement is needed to enhance the model’s sensitivity to subtler emotional cues. While the reported accuracies (95–98%) across the Rajini Fan, Stanford Twitter140, and IMDB datasets are promising, they may partly reflect the relatively small sample sizes and single-run evaluations used in this study. Future work will incorporate multiple random seeds and larger-scale datasets to verify the stability and generalizability of these results.

The 7% increase in phrase difficulty classification accuracy underscores the importance of data quality and structured learning. Our preprocessing pipeline, particularly the entropy-based sorting, acts as a form of *curriculum learning*. By calculating the information entropy H(x) of sentences, we quantitatively rank them by complexity. Feeding these pre-sorted, high-quality data batches to the GNN_MEHO model likely forces it to learn more robust features incrementally. The model first learns from simpler patterns before tackling more complex, information-dense sentences, leading to improved generalization on the test set. This technique is especially potent for noisy and informal X data, as it helps mitigate the issues of short text, grammatical errors, and special characters by emphasizing semantic richness.

As indicated in Table 7, GNN_MEHO also demonstrated strong performance on the 20% testing data from multiple datasets, including Stanford Twitter 140 and IMDB. The model achieved an average accuracy of 98.6% on the Stanford Twitter 140 dataset, highlighting the model’s robustness across different datasets. The testing results further reinforce the effectiveness of our proposed model in real-world applications.

It is important to note that the benefits of MEHO and preprocessing are synergistic. The MEHO algorithm requires a high-quality, well-structured training dataset to effectively navigate the search space and find optimal parameters. Conversely, the sophisticated GNN model optimized by MEHO is fully capable of leveraging the additional information and improved structure provided by the preprocessing stage. This combination of *data-centric* (preprocessing) and *model-centric* (MEHO optimization) innovations is the cornerstone of our framework’s superior performance.

### 5.5 Ablation study

An ablation study was conducted to quantitatively isolate the contribution of the proposed MEHO algorithm, with the results summarized in Table 10.

The results clearly demonstrate the superior efficacy of our MEHO optimizer. While the original EHO algorithm (Group 2) already provided a significant accuracy boost over the baseline GNN (+4.3%), it did so at a substantial computational cost, increasing convergence time by 25%. In contrast, our proposed MEHO (Group 3) not only achieved a far greater accuracy improvement of 6.1% over Group 2 (10.4% over Group 1), but it also reduced the convergence time back to the efficient baseline level.

Furthermore, the proposed MEHO consistently outperforms other configurations across all evaluation metrics. It achieves the highest scores in precision (94.6%), recall (94.1%), and F1-score (98.2%), indicating a balanced and robust improvement in model performance. This comprehensive enhancement across all key metrics—accuracy, precision, recall, F1-score, and convergence speed—provides strong evidence that our modifications successfully addressed the core limitation of the original EHO: premature convergence. The MEHO algorithm avoids getting trapped in local optima, enabling a more efficient and effective search for the optimal GNN parameters, which directly translates to the superior and balanced performance observed in our main results.

The results of the ablation study clearly demonstrate that the preprocessing (especially entropy-based sorting) plays a crucial role in improving model performance. By comparing the GNN model with and without MEHO optimization, a significant boost was observed in accuracy and F1 score when MEHO is included. Specifically, when the preprocessing module is removed, the accuracy of the GNN drops by approximately 4%, while removing MEHO optimization also leads to a notable decline in both convergence speed and accuracy. Thus, both preprocessing and MEHO optimization are essential to the model’s success.

### 5.6 Limitations and future work

While the MEHO algorithm significantly enhances accuracy and convergence speed, its iterative nature introduces higher computational overhead compared to standard optimizers. Future work will focus on accelerating MEHO’s convergence speed to better accommodate ultra-large-scale feature sets. Additionally, although the current study focuses on textual data, extending the framework to multi-modal sentiment analysis, including audio and visual features, may further improve model performance in complex real-world settings.

## 6 Conclusion

Emotions play a crucial role in communicating an individual’s sentiments. Social media platforms have become a vast repository for emotional expression, as users frequently share their thoughts and opinions on various topics. This data can be extracted for emotion identification. Emotion recognition has diverse applications, including the identification of psychological disorders such as anxiety and depression, assessing the collective mood within a community, and predicting trends in the stock market, product sales, and film reception. This paper presents an advantageous approach for real-time emotion recognition on X data. It utilizes a GNN combined with a modified EHO algorithm, called GNN_MEHO. The model incorporates multiclass classification and semantic analysis techniques to accurately classify emotions, with an emphasis on computing efficiency for large-scale datasets. The system automatically handles data management and parallelization tasks, allowing users to focus on designing prediction algorithms. The MEHO algorithm significantly improves classification accuracy and convergence speed in sentiment analysis tasks. By optimizing the GNN model, MEHO not only improves accuracy but also effectively prevents premature convergence, especially in large-scale data processing.

Future research directions include the optimization of the MEHO algorithm’s convergence speed for ultra-large-scale datasets and the refinement of its ability to efficiently process the large-scale data commonly encountered in real-time applications like social media monitoring. Additionally, we will explore integrating multi-modal data, such as voice, video, and subject-specific information, to enhance sentiment analysis. This would allow the model to better understand emotions conveyed through audio and visual features, broadening its ability to interpret diverse emotional expressions. Finally, the system’s scalability will be tested on larger real-world datasets, with cloud-based infrastructure used for distributed data processing, enabling global real-time sentiment analysis. The goal is to ensure efficiency and accuracy across various practical applications, from marketing to social media monitoring. Overall, future work will expand the model’s capabilities, making it more versatile and applicable to a wider range of emotion and sentiment analysis tasks.

## Data Availability

The original contributions presented in the study are included in the article/supplementary material, further inquiries can be directed to the corresponding author.

## Footnotes

^1^ https://www.kaggle.com/datasets/atulanandjha/imdb-50k-movie-reviews-test-your-bert

^2^ https://www.kaggle.com/datasets/kazanova/sentiment140
